# Granulation of Silicon Nitride Powders by Spray Drying: A Review

**DOI:** 10.3390/ma15144999

**Published:** 2022-07-18

**Authors:** Sergey N. Grigoriev, Thet Naing Soe, Alexander Malakhinsky, Islamutdin Makhadilov, Vadim Romanov, Ekaterina Kuznetsova, Anton Smirnov, Pavel Podrabinnik, Roman Khmyrov, Nestor Washington Solís Pinargote, Alexandra Yu. Kurmysheva

**Affiliations:** 1 Laboratory of Electric Current Assisted Sintering Technologies, Moscow State University of Technology “STANKIN”, Vadkovsky per. 1, 127055 Moscow, Russia; s.grigoriev@stankin.ru (S.N.G.); a.smirnov@stankin.ru (A.S.); p.podrabinnik@stankin.ru (P.P.); 2 Department of High-Efficiency Machining Technologies, Moscow State University of Technology “STANKIN”, Vadkovsky per. 1, 127055 Moscow, Russia; kothetnaingsoe6151@gmail.com (T.N.S.); m.a_p@bk.ru (A.M.); makhadilov1@yandex.ru (I.M.); v.rom07@yandex.ru (V.R.); e.kuznetsova@stankin.ru (E.K.); r.khmyrov@stankin.ru (R.K.)

**Keywords:** spray drying, silicon nitride, powders, dispersant, binder, solvent

## Abstract

Spray drying is a widely used method of converting liquid material (aqueous or organic solutions, emulsions and suspensions) into a dry powder. Good flowability, narrow size distribution, and controllable morphology are inherent in powders produced by spray drying. This review considers the granulation factors that influence the final properties of the silicon nitride dried powders. The first group includes the types of atomizers, manifolds, and drying chamber configurations. The process parameters fall into the second group and include the following: inlet temperature, atomizing air flow, feed flow rate, drying gas flow rate, outlet temperature, and drying time. Finally, the last group, feedstock parameters, includes many factors such as feed surface tension, feed viscosity, solvent type, solid particle concentration, and additives. Given the large number of factors affecting morphology, particle size and moisture, optimizing the spray drying process is usually achieved by the “trial and error” approach. Nevertheless, some factors such as the effect of a solvent, dispersant, binder, and sintering additives considered in the literature that affect the Si_3_N_4_ granulation process were reviewed in the work. By summarizing the data available on silicon nitride powder production, the authors attempt to tackle the problem of its emerging demand in science and industry.

## 1. Introduction

Silicon nitride (Si_3_N_4_) is an advanced ceramic material that has strong oxidation resistance, excellent flexural and compressive strengths, high hardness, relatively high fracture toughness, very low friction coefficient, and good thermal properties at both room and elevated temperatures [1,2,3,4,5].

Si_3_N_4_ finds application both in the form of porous and dense ceramics [6]. Due to their high densification, high-temperature stability, good corrosive resistance, and relatively high elastic modulus, dense Si_3_N_4_ ceramics are widely used for the production of gas turbine engines, high-speed cutting tools [7,8], extrusion heads [9], rocket fairings, satellite antennas, aerospace devices, as well as bearing and sealing [10,11]. On the other hand, the combination of lightweight and mechanical properties makes porous Si_3_N_4_ highly demanded separation membranes, radomes, catalyst supports, gas filters, and other engineering applications. Moreover, this kind of material is good at moisture absorption reducing thermal and electrical conductivity [12,13]. Recently, highly-porous Si_3_N_4_ ceramics were successfully introduced to medicine as spinal fusion implants providing the desired balance between strength, toughness, biocompatibility, and favorable imaging [14]. The advantages of silicon nitride over other ceramics include its significantly higher impact strength and ability to withstand rapid temperature changes [15]. However, compared to Al_2_O_3_ or SiC, Si_3_N_4_ material is more expensive [16]. The production process is rather labor-consuming due to the low self-diffusivity of this covalent material [17]. The most used crystallographic structures in industry are α- and β-Si_3_N_4_, which have their own intrinsic properties [18]. The α to β phase transformation takes place at high temperature when α particles dissolve into a liquid phase and precipitate as β phase [19]. Of these two crystal structures, the β phase is the more stable having an elongated rod-shaped structure, which allows it to act as a reinforcing phase that improves the materials’ strength [20].

At present, Si_3_N_4_-based parts can be obtained by various methods, such as direct coagulation casting [21,22], reaction sintering [22], spark plasma sintering [23,24,25], tape casting [26], hot-press sintering [27], gel casting [28,29], and others.

Due to the strong Si–N covalent bond, silicon nitride particles have low sintering activity and poor compactability, which is usually overcome by adding sintering aids in order to obtain dense microstructures of silicon nitride [30,31,32]. The sintering aids promotes the formation of a liquid phase at a relatively low temperature, which leads to greater compaction of Si_3_N_4_, supporting the grains’ reorganization and the deposition process of β phase. As a result of this process, densified Si_3_N_4_ ceramic primarily consists of grains of β-Si_3_N_4_ and glass phase obtained from the liquid phase. Oxides of rare earth, such as Y_2_O_3_, Yb_2_O_3,_ and Sc_2_O_3_, as well as metal oxides-MgO or Al_2_O_3_, are frequently used as sintering aids for silicon nitride [33,34,35,36]. Besides, MgO, Al_2_O_3_, and Y_2_O_3_ are used to form phases such as SiAlON, MgSiO_4_, and MgSiO_3_ [37,38]. In addition to the listed materials, other additives such as MgSiN_2_, YbF_3_, and Y_2_Si_4_N_6_C have been proposed to increase the nitrogen/oxygen ratio [33,39]. Furthermore, Tatami et al. [40] reported that a joint addition of AlN and TiO_2_ with sintering aids resulting in TiN microstructures in Si_3_N_4_ ceramics improves the fracture toughness the sintered ceramics.

An improvement in mechanical properties of sintered ceramics and composites can be achieved through the grain size control, in which the smaller the constituent phases and the better distributed they are in the matrix, the greater the improvement. Therefore, to obtain ceramic powders of small sizes with a morphology that will ensure the best distribution during the material loading process is crucial in order to produce Si_3_N_4_-based parts with improved mechanical properties.

Spray drying is one of the most popular methods in the large-scale production of rounded particles with a narrow size distribution due to its affordability, performance, and cost-effectiveness, in which the composition and morphology of the resulting particles can be controlled [41]. Spray drying is a method in which a liquid is brought into powder form. It is widely used in the food industry to produce freeze-dried products such as milk powder. Spray drying is also used in the pharmaceutical industry for the dehydration of saturated solutions and the isolation of the active substances contained in them in powder form [42]. Finally, this method is used for the manufacture of spherical non-agglomerated and monodisperse metastable ceramic powders and their compositions [43]. The drying particles obtained after spraying are often referred to as microspheres, spherical powders, grains, granular powders and granules, which are the terms used in the scientific literature. Generally, ceramic microspheres can be classified into two large groups: the first one is defined as dense and smooth granules with high apparent density, strong compatibility, and high surface area, while the second group is defined as highly porous or hollow granules with a high surface area [44]. The microspheres that belong to the first group are commonly used to make dense and transparent ceramics, while the rest are suitable for catalysis, drug delivery systems, and sorbents. Dense and smooth spherical powders are often used in additives technologies such as laser [45] and cold spray manufacturing [46], even with the use of ceramic materials [47].

**Figure 1:**
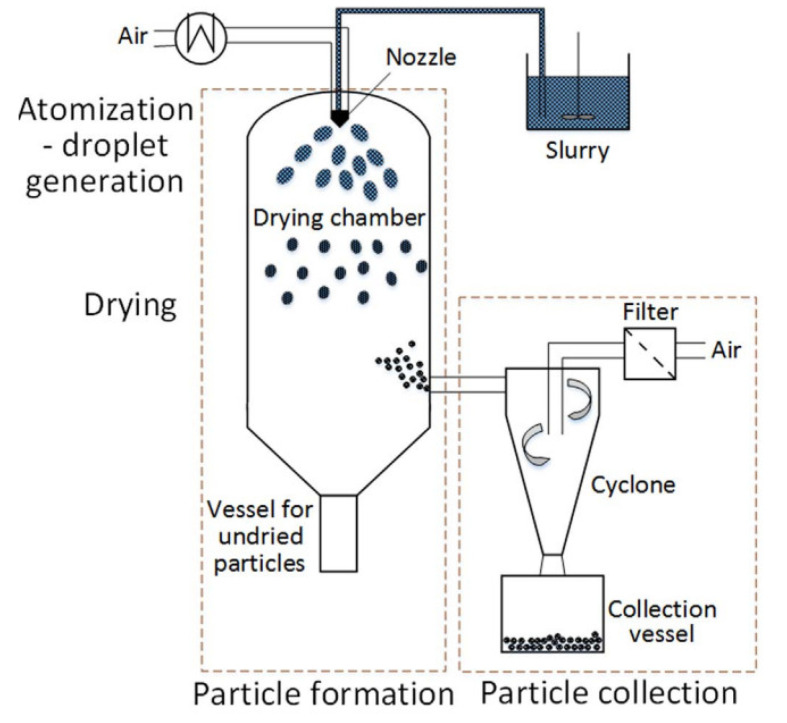
Diagram of a common laboratory scale spray dryer. Droplets and granules are depicted oversized for better visibility. Reprinted from [49].

The spray-drying process consists in converting an initial aqueous suspension (slurry for ceramic production) into a dry powder by spraying it in a heated medium such as air or inert gas. This process is generally outlined in three steps: droplet generation (atomization or spraying); droplet-to-particle conversion by dehydration (solvent evaporation); and particle collection (separation from drying gas) (Figure 1). In the first step, the suspension is pumped into the atomizer located in the drying chamber. Thus, a large number of droplets are formed and, due to surface tension, these droplets quickly become spherical. In the second step, a rapid evaporation of the aqueous medium takes place due to the high droplets’ surface area to volume ratio. In the last step, the resulting dry powder is separated from the hot air and enters the collector for further processing [48].

The explained atomization process can be carried out using various types of atomizers based on the application of pressure, centrifugal force, and ultrasonic or electrostatic energy. These atomizers are used depending on the type of initial solution, as well as on the requirements for the final size, structure and shape of the resulting product [50]. Among the atomizers, it is possible to find rotary atomizers and atomizers with pneumatic or hydraulic nozzles. Despite the variety of atomizer designs, chamber geometries, and collector types, the common parameters that mainly control the spray dryer process are inlet temperature, feed flow rate, atomized air flow (atomization pressure), drying gas flow rate, and drying time. These parameters directly affect the temperature, final humidity, yield, and size of the final particle. Moreover, feed viscosity and feed surface tension directly influence the granulating process and the final properties of the granules. As is well-known, these two suspension characteristics are affected by parameters, such as solvent properties, particle properties, additives, and constituent concentration.

The objective of this review is to make a summary of the available information related to the silicon nitride granulation process by spray drying, to perform an analysis of the factors that directly influence the final properties of the obtained powders, and show new methods for obtaining these ceramic structures.

## 2. Granulation Factors

Due to the multitude of factors to consider for spray dry granulation, this process tends to be very complicated. In this way, different parameter combinations for a suspension can provide granules with different morphologies, sizes, or amounts of residual moisture. Therefore, it is critical to understand how these parameters influence the granulation by spray-drying to achieve an optimized process.

As stated in the Introduction, inlet temperature, feed flow rate, atomized air flow (atomization pressure), drying gas flow rate, and drying time are the controlled parameters that mainly manage the spray dryer process. However, in addition to these, there are three additional parameters (equipment, process, and feedstock) that influence dry granule properties. Beneath, we will consider each of them in a summarized form.

### 2.1. Equipment Parameters

The equipment parameters group includes atomizer and collector types, as well as the drying chamber configurations.

In the start of the granulation process, the suspension is pumped into the atomizer and then a large number of droplets are formed and, due to surface tension, these droplets quickly become spherical. Different types of atomizers in spray drying can be used based on the application of pressure, centrifugal force, ultrasonic or electrostatic energy [51]. The most common ones are pneumatic nozzle, hydraulic nozzle, and rotary atomizers. The choice of atomizer type depends on the initial solution, as well as on the requirements for the final size, structure and shape of the resulting product [50].

The rapid evaporation of the aqueous medium from the droplets is carried out in the drying chamber due to the high ratio between the surface area and the volume of the droplets and the use of hot air. The shape and size of the drying chamber also play an important role in the granulation process, but these parameters are difficult to vary and even more so in the industry. Generally, a correct combination of controlled process parameters minimizes the influence of the drying chamber.

The separation of the resulting dry powder from the hot air occurs in the collector, which consists of cyclone and collection vessel (Figure 1).

More details about the technical data, construction and configuration of the drying chamber and types of collectors can be found in published works on this topic [52,53].

### 2.2. Process Parameters

The process parameters include inlet temperature, atomized air flow (atomization pressure), feed flow rate, drying gas flow rate, outlet temperature, and drying time.

#### 2.2.1. Inlet Temperature

The temperature of the drying gas just before it enters the drying chamber is called the inlet temperature. Before entering the chamber, this gas must be heated to a certain temperature to dry the atomized droplets of the suspension. Thus, at a higher temperature, a higher rate of evaporation of the solvent is obtained. However, the inlet temperature also influences the wet-bulb temperature inside the chamber, which is the temperature reached when the gas is saturated with the vapor from the liquid. At an optimum wet bulb temperature, a constant evaporation rate can be reached, in which the droplet water diffuses rapidly from the core to its surface, providing a constant loss of moisture [54,55].

#### 2.2.2. Atomizing Air Flow

For air atomizing spray nozzles, also called two-fluid nozzles, the atomization stage is carried under compressed air or another atomizing gas, which is supplied to the atomizer. The level of atomization is mainly a function of the amount of gas used and has low dependence on the liquid pressure and the spray pattern type. Thus, the higher the airflow and pressure, the smaller the drops and the size of the granules obtained. This means that even very low flow rates at low fluid pressures can be finely atomized [56].

#### 2.2.3. Feed Flow Rate

Feed flow rate also influences the granules size. The feedstock solution is supplied to the atomizer by a pump at a controllable rate. An increase in feed flow rates means an increase in droplet size, while the atomizing pressure remains constant [54].

#### 2.2.4. Drying Gas Flow Rate

The drying gas flow rate is the volume of drying gas that is supplied to the drying chamber per unit time. The application of high drying gas flow rates minimizes the air-droplet interaction time thanks to the increase of the particles’ movement inside the chamber, which prevents the complete moisture removal from the particles and leads to their agglomeration in the cyclone and the collector. On the other hand, the higher the drying gas flow rate, the higher the efficiency during cyclone separation. Thereby, the drying gas flow rate must be high enough to ensure the high efficiency of the separation process and low enough to provide complete removal of moisture from the particles.

#### 2.2.5. Outlet Temperature

The outlet temperature is the temperature of the drying gas together with the dry particles before entering the collection system. Generally, its value should be equal to or less than the maximum temperature to which the particles can be heated, but above the evaporation point of the solvent used. This temperature is not regulated by the operator and is reached thanks to the combination of certain parameters such as the inlet temperature, the drying gas flow rate, the feed flow rate, the feed solid particles concentration, and the solvent evaporation temperature.

#### 2.2.6. Drying Time

The drying time is one of the factors that directly influence the final product. It indicates time interval spray drops are inside the drying chamber. To guarantee to obtain a dry product, the drying time must be long enough, but bearing in mind that the longer the drying time, the greater the probability of thermal degradation of the material to be dried, especially in thermosensitive materials.

### 2.3. Feedstock Parameters

The rheological and interfacial properties of the feedstock are critical properties that have a great influence on the dry particles’ final characteristics, such as residual moisture, particle size, and morphology. Besides, the rheology and surface tension of the feedstock play an important role during the droplet break-up phase and establishment of desired particle morphologies, and it is necessary to know how these parameters influence the granulation process. Moreover, it is necessary to take into account that certain properties of the feedstock, for example, the abrasive properties, flammability, and pH factor, must be considered and controlled to provide the safety of the operator and the equipment to be used.

Parameters such as solvent properties, particle properties, additives and constituent concentration have a direct influence on the viscosity and surface tension of the feed and will be discussed briefly.

#### 2.3.1. Feed Surface Tension

The formation of drops from the feedstock occurs thanks to the ease of altering its surface tension during the spraying process. This means that a suspension with a high surface tension makes the fine droplet formation process more difficult since it requires more energy to generate an additional interface. If the applied energy is less than needed, then much larger droplets will be formed or, in the worst case, the atomization process will not take place. As will be seen further, during the preparation of the suspension certain additives are used to reduce surface tension.

#### 2.3.2. Feed Viscosity

Feed viscosity is a viscosity of the suspension under controlled shear strain rate in the atomizing device. Determining flowability of the feed in the spray dryer system, feed viscosity is one of the main parameters affecting drops’ size during the atomization process. However, using suspensions with viscosity increasing directly proportional to shear strain rate (i.e., dilating suspensions) provokes problem of preventing proper droplet formation and limiting the maximum strain rate value. Solutions with high viscosity values require a higher energy input to provide the given volume flow rates in the atomizer.

Feed viscosity depends on its molecular structure, molecular weight and temperature, and can be determined by the solvent type, solid particle properties, the presence of additives, the constituent’s volumetric fraction and the interaction between each of them.

#### 2.3.3. Solvent Type

**Figure 2:**
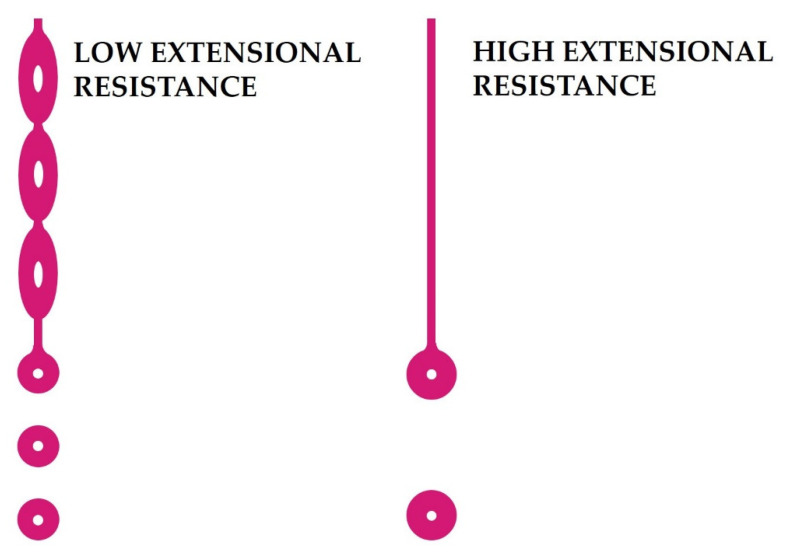
Products with high extensional resistance inhibits droplet pinch-off, tending to result in larger drop sizes. Reprinted from [58].

The solvent type (organic or inorganic) plays an important role in the preparation of the suspension with adequate viscosity for the granulation process, since the liquid phase is responsible for powder suspension, providing fluidity and dissolving the remaining additives. There are two types of solvents: carbon-free or inorganic solvents such as water and ammonia, and organic solvents such as alcohols and glycol ethers [57]. Water is one of the most widespread solvents due to its accessibility, low cost, environmental friendliness and high safety as it is neither flammable nor explosive. However, sometimes it becomes necessary to use solvents that can be flammable, prone to explosion, or have a high pH factor. In such cases, appropriate precautions must be taken to prevent damage to the operator’s health and the spray drying system. Regardless of the solvent type used, it must provide low resistance to extension to achieve the deformation necessary for “pinch-off” into smaller droplets (Figure 2).

#### 2.3.4. Particle Properties

The particle size, particle distribution, morphology (particle shape) and manufacturing process are the particle properties, which affect the suspension behavior [59] and contribute to viscosity and the granulation process. Furthermore, it should be noted that abrasive containing feedstock provokes equipment wear degrading the spray system, pumps, pipes, walls, etc.

#### 2.3.5. Solid Particle Concentration

The amount of solid present in the feedstock has a great influence on the product performance. In particular, it could be concluded whether the feedstock will be dried successfully or if there will be residual moisture. Moreover, high content of solid particles means the higher production rate, but with a slower drying rate of the feedstock.

#### 2.3.6. Additives

Generally, for the feedstock preparation, different components such as solvents, solid particles, and additives are used. Among the additives are surfactants, plasticizers, binders, and dispersants, where each of them has a specific role and each one can affect rheology behavior. Surfactants are used to control the surface tension of the feedstock, thus allowing for better wetting. The plasticizer and binder are added to hold the particles together and help maintain the shape of the granules until the completion of the subsequent pre-sintering process. Plasticizers and binders do not increase the viscosity of the system much since it limits the maximum number of solid particles that can be present in the dispersion. Dispersants are used to obtain a more stable suspension, and to reduce feedstock viscosity, thus allowing more solid particles to be added to it [60].

## 3. Influence of Feedstock Parameters on Si_3_N_4_ Granulation Process

As indicated in Section 2, different factors affect the granulation process of any suspension. Among these factors, the feedstock parameters such as the solvent type, the additives, the particle properties, and their concentration are the factors that most affect the quality and final properties of dry granular powders.

In the case of silicon nitride, due to its low level of sintering, sintering aids are added to the main components in the feed formation stage.

The current section attempts to systematize data available on the granulation process of silicon nitride according to the factors that influence the greatest on the process: solvent type, particle properties, solid particle concentration, and additives.

### 3.1. Solvent Type

The shrinkage process of atomized droplets due to solvent evaporation is an important stage in spray drying. This is also related to the silicon nitride granulation process.

Most research in silicon nitride granulation have been carried out using distilled water as the solvent (Table 1). For instance, Wu et al. [36] published a patent, in which water was used as solvent. The authors claimed a Spray drying granulation method of silicon nitride ceramic powder used for mechanical sealing, which is characterized in that the method comprises the following steps: weighing powder prepared by the following components by weight; proportioning sintering aid slurry; proportioning a binder and an antifoaming agent solution; proportioning silicon nitride slurry; proportioning silicon nitride mixed slurry; obtaining silicon nitride powder with different particle size ranges. The method is high in yield, low in production cost, safe for production, low in environmental pollution, and stable for powder performance. The present invention has the following advantages: since the employing deionized water is dispersion medium, to be mixed in certain sequence slurry by powder and a certain amount of deionized water of certain weight proportion, ball milling homogenizing and refinement, the spray-dried silicon nitride mixing granulation powder that is a granulated into, institute’s pelletizing shape particulate material has good fluidity, composition is uniformly dispersed, loose density is stabilized, adjustable, and processability is good. Thus, it is suitable for normal pressure-sintered silicon nitride continuous batch pressed compact and hot-pressed sintering silicon nitride. The present method can produce low viscous dense suspension slurry and cost is low, do not have organic solvent volatilization dangerous, and the silicon nitride powder output of preparation is high simultaneously, production cost is low, production safety, low in the pollution of the environment, properties of powder stable.

Furthermore, this table shows that there are investigations in which other solvents were used. For instance, Iijima et al. [61] have been used toluene as solvent. In this research, the effect of fatty acid structure of PEI-fatty acid complex on the dispersion properties of Si_3_N_4_-Y_2_O_3_-Al_2_O_3_-AlN-TiO_2_/toluene slurries was investigated. Moreover, the authors observed that both PEI-OA and PEI-ISA can stabilize the multi-component (Si_3_N_4_-Y_2_O_3_-Al_2_O_3_-AlN-TiO_2_) toluene slurries, which resulted in slurries possessing flow curves without hysteresis properties. In addition, it was found that PEI-ISA-stabilized slurry tended to have slightly high viscosity owing to the contact of PEI segments protruded among the short ISA chain in toluene. This weak interaction between PEI-ISA modified particles was observed to form small flocculated structures during the spray drying process, which resulted in granules having filled structures and relatively high surface roughness whereas granules obtained from PEI-OA stabilized slurry had a smoother surface and hollowed structure.

In the patent of Cui et al. [62] ethanol was used as solvent in order to obtain slurry that can be used for granulation of silicon nitride. According to the patent description, the ceramic slurry comprises the silicon nitride powder, a sintering aid, and an organic component having a hydroxyl group, a carboxyl group, an amino group, an ester group, an aldehyde group, a carbonyl group, and other groups. The obtained silicon nitride ceramic slurry has an impurity content of less than 5 wt%, a solid phase volume fraction of 50–75%, a viscosity of less than 0.2 pa s, and an α-phase content of more than 60 wt% in the silicon nitride powder, which is simple to prepare. This silicon nitride ceramic slurry has advantages of both high solid phase volume fraction (50–75%) and low viscosity (less than 0.2 pa s). Similarly, Naoto et al. [63] used ethanol as a solvent.

### 3.2. Particle Properties

Due to covalent bonding, silicon nitride has a high brittle-ductile transition temperature and low diffusion mobility. Therefore, obtaining silicon nitride parts with a relative density of >85% even at high sintering temperatures is rather difficult. To intensify the sintering of Si_3_N_4_ powders, oxide additions as sintering aids (Y_2_O_3_, Al_2_O_3_, MgO, CeO_2_, La_2_O_3_, and others) are used in an amount of 5–25% [36,61,62,63,67,69,71,73]. During sintering, these oxides interact with SiO_2_ and oxynitrides, which are formed on the surface of silicon nitride particles during their synthesis, and together form a liquid phase that enhances sintering of Si_3_N_4_-based materials [76,77].

In addition to intensifying the sintering process, the right choice and a correct combination of different sintering aids can improve the mechanical, tribological and electrical properties of silicon nitride-based materials. For instance, Gábrišová et al. [78] showed that Si_3_N_4_ with Al_2_O_3_ + Y_2_O_3_ sintering aids (YAG) in comparison to Si_3_N_4_–MgO has several times greater wear resistance. Furthermore, sintering additives MgO and Al_2_O_3_ + Y_2_O_3_ have influence on the type of crack indentation with Vickers indenter. For the ceramic with MgO sintering aid the indentation crack is of Palmqvist’s type, while the indentation crack is of half-penny shape when the Al_2_O_3_ + Y_2_O_3_ sintering additive was used. In another work, Tatami et al. [40] reported that a further addition of carbon nanotubes into the Si_3_N_4_-Y_2_O_3_-Al_2_O_3_-AlN-TiO_2_ system improve the obtainment of dense ceramics based on Si_3_N_4_ with unique electrical conductivity. The powders were mixed in ethanol together with the carbon nanotubes and dispersant in a ball milling using SIALON balls.

So far, only a few works surveyed the granulation process of the Si_3_N_4_ powder by spray drying, as well as size distribution of the obtained spherical powders and its morphology. Oda et al. [70] published a method for preparing silicon nitride granules by spray drying and a process for producing sintered products of them. In their work, the authors proposed to use as sintering aid an oxide of an element of the Group 3a (Y_2_O_3_, Yb_2_O_3_, Lu_2_O_3_, Sc_2_O_3_) of periodic table having an average particle diameter of 1.0 μm. Apart from proposed additives, the authors suggested to use Si_3_O_4_, Al_2_O_3_, as well as oxides, carbides and silicides of W, Mn, Fe or Cu from 0.1 to 1 part by weight per 100 parts by weight of silicon nitride. The authors noted that the use of these additives improve the powder flowing property, as well as they stimulate the nitriding process and the sintered product exhibited improved mechanical strength and decreased dispersion in the characteristics.

The surface quality of the Si_3_N_4_ raw particles influences the viscosity of the slurry. For instance, Tsuzuki et al. [71] in their patent claimed a process to obtain Si_3_N_4_ powder synthesized by the silicon diimide decomposition process with surface acidic groups per B.E.T. surface area in the range of 0.2–2.5 μeq/m^2^ and a method for its sintering. The as obtained Si_3_N_4_ powders with 0.2 μm average particle size were mixed with Y_2_O_3_ and Al_2_O_3_ (0.25 μm, and 0.15 μm average particle size, respectively) by wet mixing. The viscosity of the suspensions varied from 540 to 1330 centipoises (CPs), with the lowest value belonging to the Si_3_N_4_ powder with the highest value of surface acid groups by B.E.T. surface area, and the higher viscosity value belonging to the powder with lower surface acid groups by B.E.T. surface area. These suspensions can be used for the manufacture of granules by spray drying.

However, far too little attention has been paid to studying the influence of sintering additives on the silicon nitride granulation process and the properties of the obtained spherical powders. Kamiya et al. [69] reported that sintering additives can reduce the viscosity of slurry with an excess of dispersant. Commonly, the excess of dispersant increases the slurry viscosity and promotes the powder agglomeration, but the presence of ultrafine sintering additives with a large specific surface area allows adsorption of the dispersant on their surface, which leads to a reduction in the viscosity of the suspension. Cui et al. [62] showed that there is a dependency between the slurry viscosity and the content of sintering additives. In this document, the authors reported that by varying the ratio of studied sintering aids (Al_2_O_3_, Y_2_O_3_, MgO, CaO, and magnesium silica nitrogen), the viscosity of the resulting suspension variated from 0.125 to 0.180 pa·s, thus demonstrating their dependence. It is necessary to point out that in the studied examples the components were mixed in a sand mill with silicon nitride grinding balls for 2 h. Thereby, the surface of the silicon nitride particles after grinding is an unoxidized surface as it is always wrapped by solvent, dispersant and other components and isolated from outside air. On the other hand, in the case of the simple mixing (no surface shaping in a sand mill) the viscosity of the mixture reached 0.974 (pa·s). This shows that the quality of the initial particle surface also has an influence on the slurry viscosity.

Park et al. [74] revealed a strength drop as the number of sintering additives increases. Along with that, the flowability of the powder obtained lowered as well. The findings, according to the authors, indicate that the size of a silicone agglomerate element rises due to liquid sintering as the number of sintering additives grows. In turn, it leads to inflation of interagglomerate pores.

**Figure 3:**
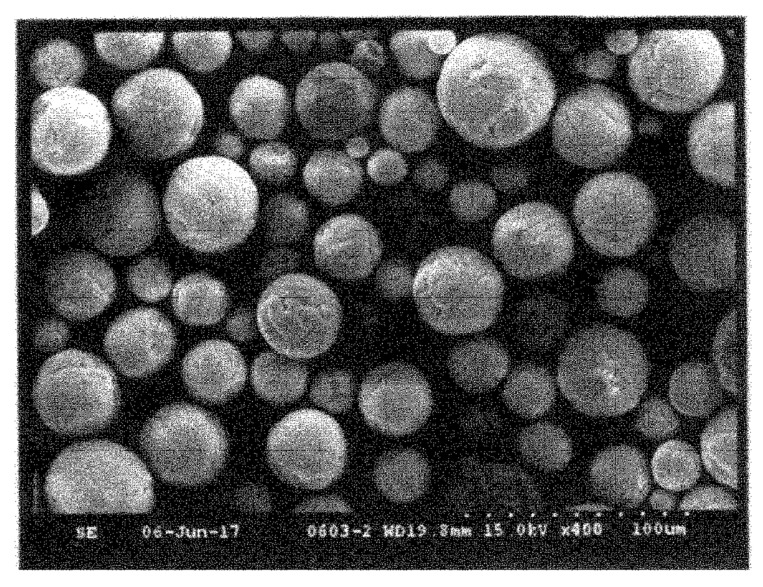
Silicon nitride granules obtained in [72].

**Figure 4:**
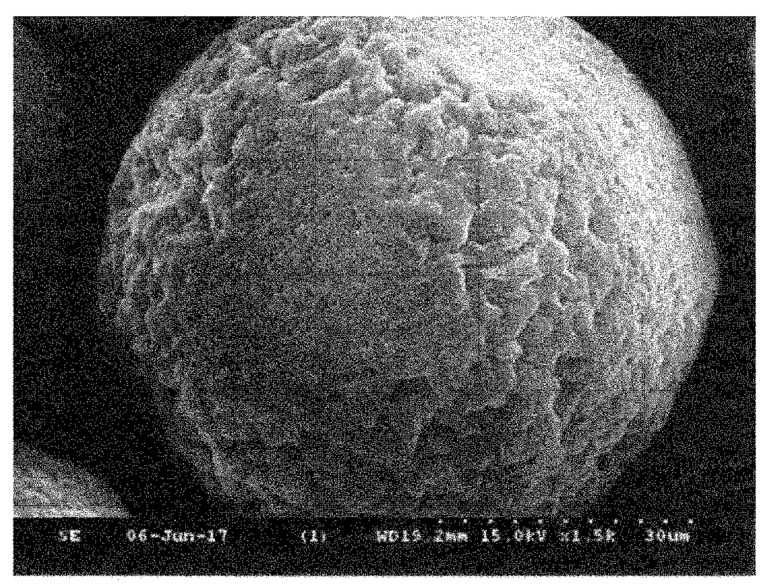
Scanning electron microscopic diagram showing a spherical powder spray granulation process produced in accordance with the embodiments of the [72].

An interesting method for producing a spherical silicon nitride powder was proposed by Wu et al. [72]. This method involves mixing source materials, spray drying, carbonization, carbothermal reduction, nitridation, and carbon removal to produce a spherical silicon nitride powder with superior characteristics. The spray granulation process is adopted to carry out the atomization process of a silicon dioxide and carbon source mixed slurry to produce a dry spherical powder. This method has the advantage of directly controlling the powder diameter within the range of 40–50 μm. The authors suggested to use as the carbon source one component of the following components: glucose, sucrose, phenol formaldehyde resin; and use as solvent deionized water or ethanol. Moreover, through changing the parameters of rising temperature curve, it is possible to complete the process of carbonization, carbothermal reduction and nitridation at the same time, for producing a spherical silicon nitride powder with high purity. Figure 3 shows the obtained powder are spherical with an average diameter of 45.36 μm. Next, the authors of the patent calcinated the obtained powder at 800 °C in a nitrogen atmosphere for 2 h, and then at 1450 °C in a nitrogen atmosphere for 5 h. The granules processed in this way are shown in Figure 4.

The Si_3_N_4_ fabricated using such method are fine powders and also has the ad-vantages of having even particle size, high purity and is capable of large-scale production. Moreover, the spherical structure of the silicon nitride powder has a superior powder fluidity, to increase bulk compactness and true density after mold formation to facilitate developing silicon nitride substrates with anti-shock and pressure resistance characteristics subsequently.

### 3.3. Solid Particle Concentration

Only a few previous studies made the effort to find an accurate ratio between the solid and liquid phases in a suspension of silicon nitride for spray drying. Available research publications have reported that the mass fraction of powder in a suspension can reach values up to ~66%. For instance, Lei et al. [75] proposed a method for the preparation of silicon nitride slurry that can be used for it granulation by spray drying. The authors affirm that the proposed method allows preparing suspensions with a content of solid phases of up to 60 wt%. The obtained granules had high product yield and product purity. In another work, Hotta et al. [65] researched the strength change of silicon nitride ceramics with the alteration of spray drying conditions. In this work, the raw materials were silicon nitride, alumina and yttria powders with an average particle size of 0.44, 0.33, and 0.29 μm, respectively. Powders were mixed by ball-milling in distilled and deionized water for 24 h. The concentration of the powder mixtures was 65.9 mass%. Dispersant was not added since the pH of the slurry moved to the basic region (~9.5), at which silicon nitride could be deflocculated electrostatically, due to the reaction of silicon nitride and water during mixing. Then, the binder (polyvinyl alcohol), plasticizer (polyethylene glycol), and lubricant (stearic acid emulsion) were added in the concentrations of 0.9, 0.45, and 0.9 mass%, respectively. The authors conclude that the differences in the fracture strength of sintered silicon nitride made from the granular compaction route could be explained quantitatively by the difference in the size distribution of pore defects in the sintered bodies. Moreover, were observed potential flaws in green compacts, that were introduced by the non-uniform packing of powder particles resulted from the incomplete collapse of dimples in granules and of interstices between granules. Formation of the potential flaws during compaction depended on the mechanical properties and the resultant compaction behavior of the granules. In [61], the authors also kept the ratio of the solid phase to the liquid equal to ~65%.

### 3.4. Additives

As indicated in Section 2, the additives used in the preparation of the feedstock are surfactants, plasticizers, binders and dispersants, but the latter two have a greater influence on the process of obtaining granules by spray drying. These two parameters will be discussed in above.

#### 3.4.1. Dispersants

In order to manufacture highly reliable ceramic parts without large pores or other defects, it is important to use Si_3_N_4_ granules with a dense and uniform structure, which largely depends on the degree of flocculation or dispersion of the slurry. In a flocculated suspension, a weak repulsive force exists between powder particles due to van der Waals forces. After drying, these flocculent particles lead to the formation of granules with a porous structure as a result of loosely packed particles. On the contrary, in a disperse suspension, the particles separate from each other, which ensures the production of dense granules [79]. According to [80], the porosity of the granules should not exceed 30%.

Silicon nitride powders contain on their surface a silica layer with a thickness of less than a nanometer, which, together with sintering aids, promotes sintering processes. More specifically, silanol (Si–OH) and secondary amino groups (Si_2_–NH) can be found on the surface, which may leach in an aqueous medium, changing the chemical composition of the surface of solution powders [81]. An increase in silanol groups intensifies the negative potential, which causes repulsion between particles, which contributes to obtaining a more dispersed suspension for slurry production [82]. Additionally, calcination steps described in [83] were used to form a thicker silica layer, and hence more silanol groups on the surface of silicon nitride particles. However, more silica results in lower mechanical properties at elevated temperatures of the sintered bodies, since they contain more intergranular quartz glass [84,85,86]. Another method for stabilizing ceramic powders is to use polyelectrolyte dispersants, consisting of low molecular weight water-soluble polymers, which, being adsorbed on silicon nitride particles, prevent powder particles from sticking together due to electrostatic repulsions [87,88].

**Figure 5:**
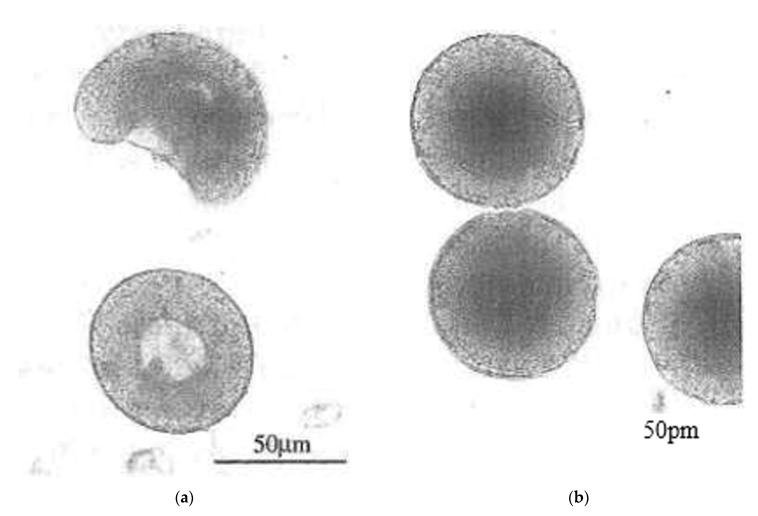
Comparison of granule structure. Dispersion state of spray-dry slurry was varied by controlling pH of slurry. (**a**) dispersed (pH = 10.8) and (**b**) flocculated (pH = 7.9). Reprinted from [66].

In the work of Takahashi et al. [66], the degree of dispersion of the suspension was controlled by changing the pH. Knowing the pH value of the isoelectric point and the ξ-potential of the surface of Si_3_N_4_, A1_2_O_3_, and Y_2_O_3_, the authors found that the electrostatic repulsion between the negatively charged regions of the surface of the suspension components ensures good dispersion in the region of pH 10.8. On the contrary, at pH 7.8, the electrostatic repulsion between particles is weak, which leads to their flocculation. The former approach resulted in particles with an irregular shape unlike spherical and uniform ones obtained from flocculated suspension (pH = 7.9). The granules obtained from a dispersed suspension (pH = 10.8) have an irregular shape, most of them have a notch (Figure 5a). Spray-dried granules from a flocculated suspension (pH = 7.9) are spherical and uniform (Figure 5b). The relatively dark appearance of the granules obtained from the flocculated suspension indicates the existence of pores in the granules, i.e., the presence of open structures [66,67].

Lukasiewicz et al. [89] showed that strong agglomeration of particles in the flocculated slurry system causes numerous micropores in the sintered body, and these micropores limit the strength of the sintered silicon nitride to about 1000 MPa. Furthermore, the authors also found that granules from a flocculated suspension are compacted and break during molding, while particles from a dispersed suspension are only deformed.

**Figure 6:**
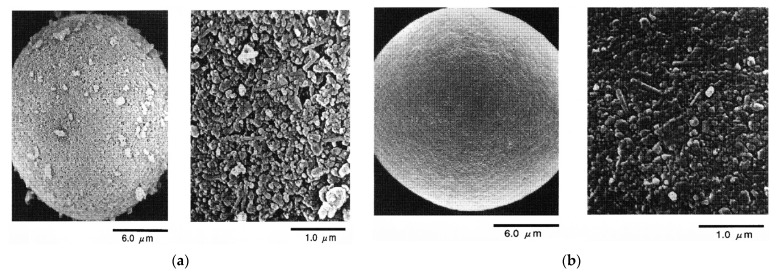
SEM observation for spray-dried granules. Sintering aid concentration 5 wt%. Concentrations of water-soluble maleic anhydride dispersant were (**a**) 0 wt% and (**b**) 2 wt%. Reprinted from [69].

Kamiya et al. [69] observed a rough surface and porous structure (Figure 6a) in the granules obtained by the absence of a dispersant, as a result of powder agglomeration in the suspension. Further, with a dispersant content being 2 wt%, the raw particles were closely packed in the granules (Figure 6b) by the capillary force created during spray drying. Moreover, the authors noticed that scanning electron microscopic (SEM) observations corresponded to the results measured by mercury porosimetry. Accordingly, the agglomerations in suspension retained the spray-dried granule structure.

However, electrostatic stabilization is often insufficient for powder systems with complex surface chemistry, such as silicon nitride [90]. In this case, steric stabilization is required to form a more stable suspension with a high content of solids [90] without changing the chemical composition of the surface and, therefore, the properties of the finally sintered parts. According to the results obtained by Yang et al. [91], steric stabilization is based mainly on two mechanisms. When two particles with adsorbed polymers approach each other, the number of conformations that the polymer can assume decreases due to the presence of the other particle. This results in a loss of conformational entropy. Secondly, the concentration of the polymer increases in the region of overlap. This results in osmotic repulsion between the particles in the suspension, which leads to stabilization of the suspension. The most common method for preparing a stable dispersed aqueous suspension of silicon nitride is the use of powder dispersants, which are mainly anionic polyelectrolytes that are adsorbed on the surface of particles after ionization and cause steric repulsion forces between particles [92,93,94,95,96].

**Figure 7:**
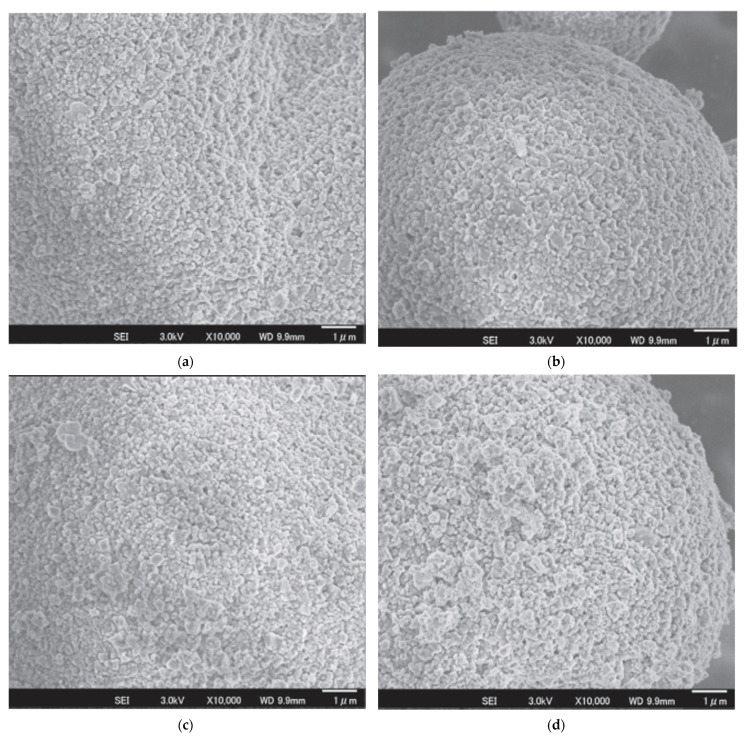
Surface structures of granules spray-dried from Si_3_N_4_-Y_2_O_3_-Al_2_O_3_-AlN-TiO_2_/toluene slurries stabilized with PEI-OA ((**a**) 1.00 mg/m^3^, (**b**) 1.30 mg/m^3^, (**c**) 1.55 mg/m^3^ and PEI-ISA, (**d**) 0.80 mg/m^3^). Reprinted from [61].

Iijima et al. [61] used as dispersants complexes of a cationic polymer of polyethyleneimine (PEI) with anionic oleic acid (PEI-OA) and PEI with isostearic acid (PEI-ISA). The initial material for obtaining granules was a suspension of Si_3_N_4_-Y_2_O_3_-Al_2_O_3_ AlN-TiO_2_/toluene. It has been found that both PEI-OA and PEI-ISA can stabilize Si_3_N_4_-Y_2_O_3_-Al_2_O_3_ AlN-TiO_2_/toluene slurries. However, the PEI-ISA slurry had a slightly higher viscosity. Compared with the PEI-OA system, the slurry viscosity was slightly higher and a slight increase in viscosity was also indicated by the existence of free PEI-ISA. Although the adsorption of PEI-ISA and PEI-OA can improve the wettability with toluene, it is expected that the loops and/or tails of PEI segments may protrude among the ISA chain owing to its short and branched structure compared with OA. These protruded hydrophilic PEI segments from different particles may be in contact with hydrophobic toluene, subsequently interacting with each other (through hydrogen bonding, for instance) and resulting in a slight increase in slurry viscosity. The spray-dried microspheres from the PEI-ISA stabilized slurry had a denser structure with higher surface roughness (Figure 7a–c). compared to the microspheres prepared from the PEI-OA stabilized slurry (Figure 7d).

In addition, it has also been observed that microspheres made from the PEI-ISA system tend to have lower densification compared to the PEI-OA ones. However, the relative density of the sintered material was higher for the PEI-ISA system, which was mainly due to the more uniform structure of the green compacts without large pores and the packing density distribution.

Interparticle forces and resultant granules’ properties can be effectively manipulated by adapting the properties of the slurry, especially the level of flocculation.

A link between the formation of hollow granules and the level of flocculation of the slurry was for the first time discussed by Lee et al. [97]. The authors have found that homogeneous granules form at low dispersant levels for non-aqueous silicon nitride slurries with PVB binder and PEG plasticizer, but hollow granules form when a high dispersant level is used. It was also found that the particle-packing density in homogeneous granules is lower than in the hollow granules. Takahashi et al. [67] have reported similar results in aqueous silicon nitride slurries when the level of flocculation is controlled by varying the slurry pH value. The pH-dependent zeta potential controls the slurry flocculation through its dominant effect on the electrostatic repulsion between the particles.

Another type of dispersant has been reported by Cui et al. [62], where the authors used chemical compounds such as triethyl phosphate, polyvinylpyrrolidone, ammonium polyacrylate, and ammonium citrate. The dispersants were added to the initial powder in a ratio of 2–4:100 g. Furthermore, the suspension was obtained using ethylene glycol, isopropanol, n-butanol and 2-butanol solvents excluding water-based ones.

Wu et al. [36] used as dispersing agent a mixture of ammonia water, tetramethylammonium hydroxide and sodium cetylsulfonate. The obtained spherical powders have good fluidity, uniform composition dispersion, stable and adjustable loose density, and good molding performance, suitable for normal pressure sintering of silicon nitride.

According to the literature, normally the amount of the added dispersant varies from 0.001% to 4.0% by weight of the initial powder in suspension [63,66,67,74].

#### 3.4.2. Binder

The criteria for choosing a binder used in the spray drying of raw materials for ceramic production are based on its ability to form granules that are easily deformed during pressing, burn out cleanly before sintering, and impart high density and strength to the compact [50]. The binder can increase the viscosity of the suspension, resulting in larger granules. However, excessive use of binders can also lead to the formation of a low permeability flexible shell around the droplet, which reduces the rate of solvent evaporation and the solvent begins to evaporate inside the granule. This situation, in turn, leads to the formation of donut-shaped granules instead of spheres [50].

During spray drying, the binder may migrate to the surface of the granules, especially if it is water-soluble. Therefore, it becomes saturated with a binder, which makes it more rigid and poorly deformable [98]. The binders commonly used for powder pressing are polyvinyl alcohol, PVA and polyethylene glycol (PEG). PEG is a low molecular weight compound made from polymerized ethylene oxide with good lubricating and plasticizing properties. The advantage of using PEG lies in its ability to achieve sufficient deformation of the granules without the addition of a plasticizer [68].

However, being hygroscopic, PEG is sensitive to changes in relative humidity, which affects the friction behavior and adhesion of the dried granules [99,100]. Shinohara et al. [101] showed that high relative humidity softens the PEG significantly reduces the flowability of the resulting powder. Low humidity values, on the contrary, resulted in harder and poorly deformable dried granules.

Granules behave similarly to elastic spheres up to a certain critical level of stress, at which they either plastically deform or break (depending on the binder concentration) [102]. The tensile strength of silicon nitride pellets should be as low as possible to ensure a narrow pore size distribution in the green body [69]. Thus, soft deformable granules improve packing by filling the intergranular pores. However, in this case, high friction forces can lead to the formation of low-density regions with incomplete deformation of the granules inside the compact [103]. Meurk et al. [68] reported that the higher the concentration of the binder and the relative humidity of the air, the more prone the resulting Si_3_N_4_ granules are to deformation than to destruction, which leads to their better compaction. On the other hand, by reducing the coefficient of friction and increasing adhesion, they can stick to the walls of the spray dryer.

**Figure 8:**
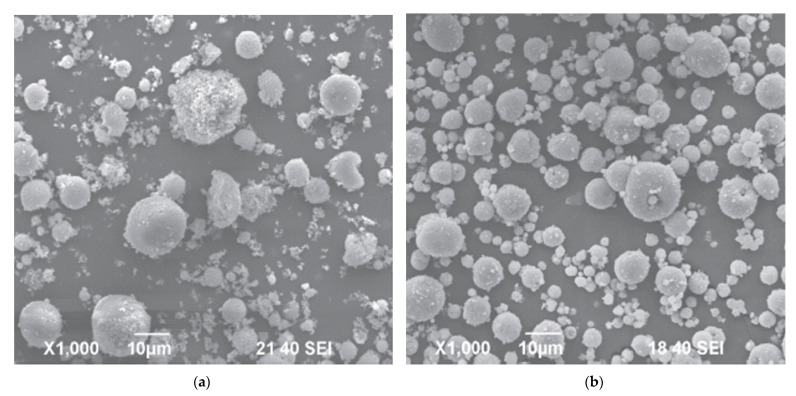
SEM image of spray-dried granules from PEI-OA (1.30 mg/m^2^) stabilized Si_3_N_4_-Y_2_O_3_-Al_2_O_3_-AlN-TiO_2_/toluene slurries using (**a**) paraffin and (**b**) PMAO as binders. Reprinted from [61].

Another work on spray-dried granules from multicomponent Si_3_N_4_ suspensions undertaken by Iijima et al. [61] was based on two types of binders capable of dissolving in non-aqueous solvent (toluene) and increasing the strength of the granules. Figure 8 shows an example of SEM images of spray-dried pellets from stabilized PEI-OA (additive content: 1.30 mg/m2) multi-component toluene suspensions using liquid paraffin and Poly (maleic anhydridealt-1-octadecene) (PMAO) as binders. The latter approach was found to be beneficial as it provided stable and strong particles while using paraffin-based binders resulted in brittle granules hence unadaptable for handling. This result suggests that the maleic anhydride segment of PMAO reacted with free amines of PEI-OA and resulted in the binding of reagent particles.

According to the present review, the addition of a polymer binder to ceramic powders before pressing them significantly improves the properties of the resulting green body. Particularly, the binder increases the cohesion of the powder and hence the strength of the green body [104]. However, it is also known that the addition of a binder can potentially lead to a large porosity of the ceramic after the sintering step, during which all organic content is removed.

In their work, Cui et al. [62] used polyethylene glycol, polyvinyl butyral and methyl acrylate as a binder. The weight content of the binder ranged from 5–20 g per 100 g of silicon nitride powder. In addition, plasticizers such as phthalate, polyethylene glycol and glycerin can be used together with the binder.

Oda et al. [70] used organic resins such as oil resin or mountain wax as binders. It was recommended that viscosity of such resins must be less than 10.6 Pa·s at temperature of 80 °C. The organic binder was admixed in an amount of 4 to 25 parts by weight per 100 parts by weight of a mixture of silicon nitride powder, silicon powder and additive powder.

Wu et al. [36] used a binder, which was a mixture of polyvinyl alcohol, dextrin, methylcellulose, and glucose. It is also mentioned that general suspension contained polyethylene glycol as a plasticizer and ethanol solvent.

Collins et al. [73] managed to obtain granules sized 2–50 μm without binders. For the production of the obtained suspensions its viscosity must to be from 150 to 500 cps viscous.

Thus, the optimal amount of binder is determined by a balance between the increase in the strength of the green material and the loss of density. In addition, it is well known that the spatial distribution of the binder in the granules can strongly influence the properties of the green body and sintered ceramic. It is well established that during the spray drying process, the binder can segregate to the surface of the granule and further form a layer at that surface. Evidence for that layer has been provided [105,106] and it has been shown to form with binders that do not (or only poorly) adsorb onto the surfaces of the ceramic colloidal particle before spray drying. Segregated layers are suspected to have a role in green strength by enhancing the adhesion between granules [104,107]. However, they can also be detrimental to the quality of the final ceramics: their removal leaves coarse defects, and segregated layers are at the origin of defaults in the final sintered ceramics [108]. They are also considered to affect the structure of the compacts [104]. However, further investigation is needed to understand the role of that layer during the compression step, given mechanical properties of the binder.

The weight content of the binder in the suspension is not fixed and varies according to the different investigations. For example, Meurk et al. [68] varied the content of the binder from 0.81 to 6.5% wt, while Hotta et al. [65], calculated the amount of binder on the mass of silicon nitride powder, which was 8.3% of the mass of the initial Si_3_N_4_. In the other works, such as Hirosaki et al. [63], Tsuzuki [71,74], the quantities of binder were 0.3–3.5%, 0.5%; and 2.12–2.25%mass, respectively.

## 4. Future Work

The review shows that controlling the conditions of the spray dry method makes it possible to obtain particles of various sizes and shapes, which may be an appropriate method for preparing initial materials for the range of powder technologies. For example, spark plasma sintering (SPS) utilizes powder to obtain dense ceramic objects with improved properties [109]. Moreover, applying ceramics to additive manufacturing (AM) is very promising and early steps are already taken in this field [110,111,112]. Its development requires fine powders of new materials as well, for which spray drying can be applied. Thus, future work shall be focused on obtaining Si_3_N_4_ powders for SPS, AM and other technologies and broaden the list of materials they work with.

## 5. Conclusions

Spray drying is a widely used technology that is used in various industries as well as in scientific research. Depending on the purpose and application of granular powders, their properties vary greatly. There are two groups of spray-dried granules. The first one is characterized by dense and smooth granules, high apparent density, strong compactness and fluidity of the corresponding powder and is used for obtaining densely sintered composites with improved mechanical properties. The second group includes highly porous and hollow granules with a high surface area for use in catalysis, drug delivery systems, or sorbents. It has been observed that there are a large number of parameters such as equipment, process, and feedstock that affect the efficiency of the drying process.

The main highlights of the review:

- Water is the most widespread solvent for preparing a suspension of silicon nitride for spray drying. However, depending on the purpose of obtaining silicon nitride powder, it is possible to use organic solvents such as alcohols, glycol ethers and others.
- To improve mechanical and tribological properties of silicon nitride materials it is recommended to use sintering aids, which are often oxides. Normally, Y_2_O_3_, Al_2_O_3_ aids in an amount of 5–25%wt. are added during manufacturing.
- The density of the granular powders is largely determined by the degree of the particles dispersion in the suspension. Porous granules are obtained when a flocculated suspension with a weak repulsive force between powder particles due to van der Waals forces is used. On the contrary, when the particles are separated from each other, for example, in a dispersed suspension, dense granules are obtained. The density is manageable by choosing the right type of dispersant.
- The criteria of binder selecting for spray drying of ceramics should be based on its ability to form granules that easily deformed during pressing, cleanly burnt out before sintering, providing high density and strength to the compact. Normally, polyvinyl alcohol and polyethylene glycol are used as binders.

Therefore, it is necessary to consider many factors influencing the final properties of dried products, rather than considering a few specific ones. The “trial and error” approach is normally used to obtain products with the desired characteristics and understand the relationship between all mechanisms as well as make the drying process completely predictable and controllable. After optimizing the process, it is possible to obtain sintered silicon nitride ceramics with improved mechanical properties derived from the spray-dried powders.

## Figures and Tables

**Table 1:** Slurry composition.

№ *	Si_3_N_4_ Particle Size, μm	Sintering Additives	Solvent	Dispersant	Binder	Other Additives	Refs.
1	n/d **	Al_2_O_3_, Y_2_O_3_, MgO, AlN	Deionized water	Ammonia water, tetramethylammonium hydroxide and sodium cetylsulfonate mixed in any proportion	The binder is one or more of polyvinyl alcohol, dextrin, methylcellulose and glucose mixed in any proportion	Plasticizer polyethylene glycol, Defoamer n-octanol, n-butanol and ethylene glycol	[36]
2	n/d	Y_2_O_3_, Al_2_O_3_, AlN, TiO_2_	Toluene	PEI-oleic acid (PEI-OA) polyethyleneimine -oleic acid complex	PMAO (Poly(maleic anhydride-alt-1-octadecene)) or paraffin	-	[61]
				PEI-isostearic (PEI-ISA) complex			
3	1.3–5	Y_2_O_3_, Al_2_O_3_, MgO, CaO	Ethanol	Triethyl phosphate and polyvinylpyrrolidone	Polyvinyl butyral	Polyethylene glycol and glycerol as plasticizer	[62]
4	0.3–3	Al_2_O_3_, MgO, Nd_2_O_3_, Y_2_O_3_, CaO, AlN	Ethanol	n/d	n/d	-	[63]
5	n/d	Y_2_O_3_, Al_2_O_3_, AlN	n/d	n/d	organic binder	SiO_2_ or MgO (to control the viscosity of the binder matrix)	[64]
6	0.44	Y_2_O_3_, Al_2_O_3_	Deionized water	-	polyvinyl alcohol	Plasticizer (polyethylene glycol) and lubricant (stearic acid emulsion)	[65]
7	n/d	Y_2_O_3_, Al_2_O_3_	Deionized water	2,2′,2″-Nitrilotriethanol citrate, distilled water, HNO_3_	n/d	-	[66,67]
8	n/d	Y_2_O_3_, Al_2_O_3_	Deionized water	Anionic polyelectrolyte	Poly(ethylene)glycol (PEG)	-	[68]
9	n/d	Y_2_O_3_, Al_2_O_3_	Deionized water	Maleic anhydride polymer		-	[69]
10	0.9	Y_2_O_3_, Al_2_O_3_	n/d	n/d	Oil resin or wax	-	[70]
11	≤1	Y_2_O_3_, Al_2_O_3_	Ethanol	n/d	n/d	-	[71]
12	n/d	SiO_2_	Deionized water, ethanol	A mixture of glucose, sucrose and phenol-formaldehyde resin	n/d	-	[72]
13	n/d	Y_2_O_3_	Deionized water	Ammonium polyacrylate	n/d	-	[73]
14	n/d	Y_2_O_3_, Al_2_O_3_, CaCO_3_	Deionized water	poly(acrylic acid) (PAA)	poly(vinyl alcohol)	-	[74]
15	0.1−10	n/d n/d n/d n/d n/d	Deionized water	n/d	amylopectin, glucose, polyhydric alcohol	-	[75]

* №—index number of the slurry composition examples, ** n/d—no data.

## Data Availability

The data described in this article are openly available in previous works.
